# Long-term observation of incremental response and antibodies to voltage-gated calcium channels in patients with Lambert–Eaton myasthenic syndrome: two case reports

**DOI:** 10.1186/s13256-015-0524-9

**Published:** 2015-03-14

**Authors:** Ilka Schneider, Malte E Kornhuber, Frank Hanisch

**Affiliations:** 1 Department of Neurology, Martin-Luther-University Halle-Wittenberg, Ernst-Grube-Strasse 40, D-06120 Halle (Saale), Germany; 2 Department of Neurology, Vivantes Humboldt-Klinikum, Am Nordgraben 2, D-13509 Berlin, Germany

**Keywords:** Lambert-Eaton myasthenic syndrome

## Abstract

**Introduction:**

Lambert–Eaton myasthenic syndrome is a rare autoimmune disorder of neuromuscular transmission due to the presence of antibodies to presynaptic P/Q-type voltage-gated calcium channels. The gold standard of therapy is the potassium channel blocker 3,4-diaminopyridine. To the best of our knowledge, no clinical reports have been published to date about long-term follow-up outcomes in patients who discontinued 3,4-diaminopyridine therapy. In addition, we know of no recent articles in which the natural history in patients with autoimmune-mediated Lambert–Eaton myasthenic syndrome has been addressed. In this report, we describe the cases of two such patients.

**Case presentation:**

Patient 1 was a Caucasian man who had been diagnosed at age 15 years with Lambert–Eaton myasthenic syndrome with symptoms of fluctuating muscle weakness and easy fatigability. These symptoms stabilized, and his electrophysiological parameters normalized, during treatment with a maintenance dose of 50mg/day of 3,4-diaminopyridine. After 5.5 years, however, he wished to discontinue the treatment. After that point, his electrophysiological parameters and presynaptic P/Q-type voltage-gated calcium-channel antibody titer remained stable. During the 15-year follow-up period, patient 1 reported mild exertion-induced complaints but did not feel restricted in his occupation and most daily activities. Patient 2 was a Caucasian man diagnosed at 32 years of age with a moderate limb girdle syndrome. He was treated with up to 80mg/day of 3,4-diaminopyridine. Because of the drug’s very short-lasting effect (<1 hour), however, he took it mostly irregularly (≤1×20mg/day). During the 14- year period of observation, his repetitive nerve stimulation responses and presynaptic P/Q-type voltage-gated calcium-channel antibody titer remained stable, his compound muscle action potential amplitudes were decreasing and his clinical symptoms did not deteriorate. At his last follow-up examination, patient 2 was independent in all of his daily activities.

**Conclusion:**

Some patients with autoimmune-mediated Lambert–Eaton myasthenic syndrome show a stable clinical long-term course without treatment. The benefit of each long-term therapy should be critically assessed during follow-up, and possible side effects should be balanced against the quality of life in these patients.

## Introduction

Lambert-Eaton myasthenic syndrome (LEMS) is a rare autoimmune disorder of neuromuscular transmission due to the presence of antibodies to presynaptic P/Q-type voltage-gated calcium channels (VGCC-Ab). It is characterized by increased fatigability, fluctuating limb girdle weakness and autonomic changes [1]. Repetitive nerve stimulation (RNS), the main diagnostic test for LEMS, is characterized by (1) an incremental response ≥100% after brief exercise, (2) a decremental response at low rates of stimulation and (3) a low compound muscle action potential (CMAP) amplitude at rest [1]. Symptomatic therapy formerly consisted of a lifelong potassium channel blocker—3,4-diaminopyridine (3,4-DAP) (the gold standard)—and (facultatively) immunosuppressive drugs [2]. To the best of our knowledge, no clinical reports have been published to date about long-term follow-up outcomes in patients who discontinued 3,4-DAP therapy. In addition, we know of no recent articles in which the natural history of patients with autoimmune-mediated LEMS has been addressed.

In this report, we present the long-term follow-up of two patients with autoimmune-mediated LEMS, one of whom took no 3,4-DAP (patient 1) for part of the follow-up period and the other of whom took low-dose 3,4-DAP irregularly (patient 2) for some of the follow-up period. Follow-up by pulmonary function testing, bronchoscopy and magnetic resonance imaging of the lung and mediastinum for more than 5 years did not show evidence of small cell lung cancer or thymoma, and the SOX1 Ab titer was normal in both.

## Case presentation

### Patient 1

**Figure 1:**
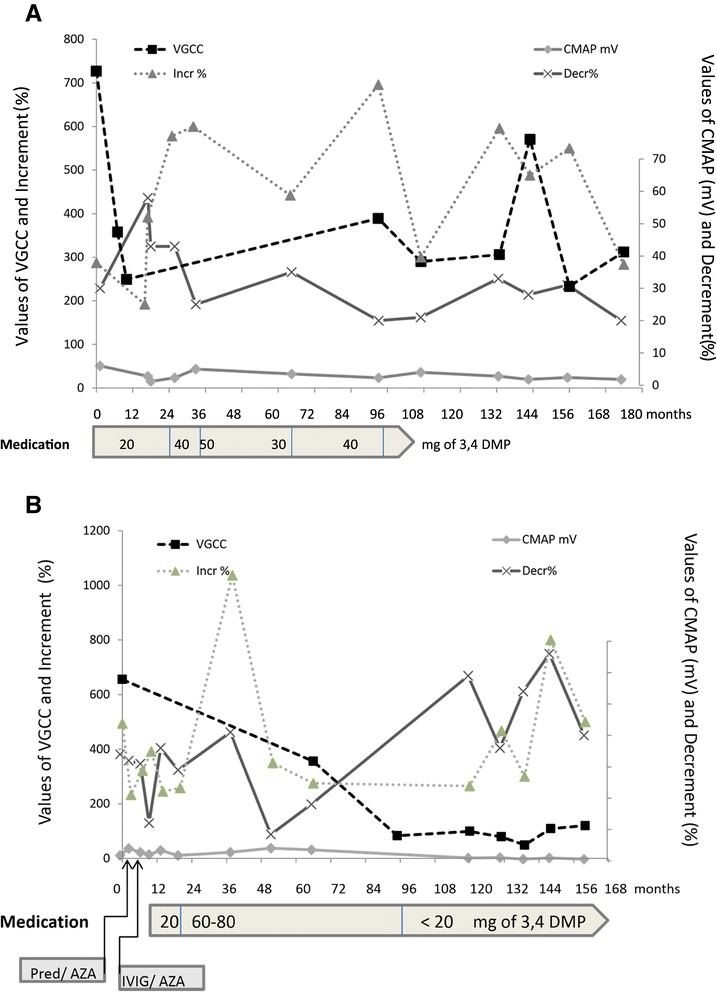
**Disease course in patients 1 and 2.** Graphs show data for antibodies to P/Q-type voltage-gated calcium channels, compound muscle action potential amplitudes, incremental and decremental response over the course of the disease in patient 1 **(A)** and patient 2 **(B)**. In both patients a repetitive nerve stimulation test was performed using the belly tendon technique over the abductor digiti minimi (supramaximal stimulation to record compound muscle action potential amplitudes at rest and after exercise for 30 seconds at 3-Hz stimulation). Mean skin temperature: 32°C; pathological decrement: ≥10% difference between first and fifth compound muscle action potential amplitudes; pathological increment: ≥100% compound muscle action potential amplitudes increase after exercise (Multiliner Vision electromyography system; VIASYS Healthcare, Höchberg, Germany). The P/Q-type voltage-gated calcium channel antibodies were analyzed by radioabsorption assay (Angela Vincent, John Radcliffe Hospital, Oxford, UK: upper limit of normal value <45pmol/L; MVZ Labor Prof. Seelig, Karlsruhe, Germany: upper limit of normal value <40pmol/L). Aza, Azathioprine; CMAP, Compound muscle action potential; 3,4 DMP, 3,4-diaminopyridine; IVIG, Intravenous immunoglobulins; Pred, Prednisolone; VGCC, Voltage-gated calcium channel.

At the time of his initial diagnosis at age 15 years, a Caucasian boy (patient 1) presented with symptoms of fluctuating muscle weakness and easy fatigability (Figure 1A). His neurological examination revealed mild proximal paresis. His physical status stabilized after treatment with 3,4-DAP was initiated (initial dose of 20mg/day, maintenance dose of 40mg/day to 50mg/day). Five and one-half years later, however, the patient wished to discontinue the treatment. After that point, his electrophysiological parameters (which had not normalized with 3,4-DAP therapy) and VGCC-Ab titer remained stable. At the time of his final examination 15 years after diagnosis, he reported exertion-induced complaints (for example, climbing stairs, lifting weight >15kg) and muscle pain lasting several days after unusual physical activities. He did not feel restricted in his occupation as a technical laboratory assistant. He had no permanent paresis, no vegetative nerve involvement and no cerebellar ataxia. His Quantitative Myasthenia Gravis (QMG) Test scores were normal.

### Patient 2

At the time of diagnosis, a 32-year-old Caucasian man (patient 2) presented with symptoms of fluctuating muscle weakness and easy fatigability (Figure 1B). His physical examination showed a limb girdle syndrome predominantly in the hip girdle. Treatment with prednisolone (40mg/day, stepwise tapering) and azathioprine (150mg/day) was initiated shortly after the diagnosis. This therapy was discontinued after 12 months due to lack of response. 3,4-DAP was then started and increased to a dose of 4×20mg/day. The patient took the drug irregularly (≤1×20mg/day), however, owing to its very short-lasting effect (<1 hour). During the 14-year period of observation, his RNS responses and VGCC-Ab titer remained stable; his CMAP amplitudes were decreasing; and his clinical symptoms did not deteriorate. At his last follow-up examination, the patient was independent in all of his daily activities. He walked with crutches (maximal walking distance, 200m) and used a stair lift (for 6 years prior to the last follow-up). He reported exertion dyspnea, dry mouth and dry eyes, but no cerebellar ataxia. His QMG Test score was 10. His physical examination revealed proximal paresis (shoulder girdle, Medical Research Council (MRC) grade 4/5; hip girdle, MRC grade 2–3/5), but no atrophies.

## Discussion

In the two patients reported here, the signs and symptoms of LEMS and its functional impact on daily activities seemed to be quite stable over time and did not deteriorate. Symptomatic treatment seemed to stabilize the disease initially, but it had no sustained effect. In the long term, reported outcomes for patients with non-cancer-associated LEMS who were on immunosuppressive therapy for a median of 6 years were that 35% had normalized resting CMAP amplitudes, 58% had increments within the normal range and 52% had decreased VGCC-Ab titers [3].

## Conclusions

The benefit of each long-term therapy for patients with LEMS should be critically assessed during follow-up, and possible side effects should be balanced against the quality of life.

## Consent

Written informed consent was obtained from the patients for publication of this case report and any accompanying images. A copy of the written consent is available for review by the Editor-in-Chief of this journal.

## Abbreviations

Ab: Antibody
CMAP: Compound muscle action potential
3,4-DAP: 3,4-diaminopyridine
LEMS: Lambert-Eaton myasthenic syndrome
QMG test: Quantitative Myasthenia Gravis Test
VGCC: Voltage-gated calcium channel
